# Within-breath oscillometry for identifying exercise-induced bronchoconstriction in pediatric patients reporting symptoms with exercise

**DOI:** 10.3389/fped.2023.1324413

**Published:** 2024-01-11

**Authors:** Mario Barreto, Chiara Veneroni, Mariaclaudia Caiulo, Melania Evangelisti, Pasquale Pio Pompilio, Maria Cristina Mazzuca, Giorgia Raponi, Jacopo Pagani, Pasquale Parisi

**Affiliations:** ^1^ NESMOS Department, Pediatric Unit Sant’Andrea Hospital, Faculty of Medicine and Psychology, “Sapienza” University, Rome, Italy; ^2^ Department of Electronics, Information and Bioengineering, Politecnico di Milano University, Milano, Italy; ^3^ Restech Srl, Milan, Italy

**Keywords:** forced oscillation technique (FOT), respiratory impedance, exercise-induced bronchoconstriction (EIB), exercise-induced symptoms (EIS), spirometry, peripheral airways, asthma, children

## Abstract

**Background:**

Evaluating oscillometry parameters separately for the inspiratory and expiratory breath phases and their within-breath differences can help to identify exercise-induced bronchoconstriction (EIB) in pediatric outpatients disclosing exercise-induced symptoms (EIS).

**Aims:**

To assess the response in impedance parameters following an exercise challenge in patients reporting EIS.

**Methods:**

Sixty-eight patients reporting EIS (34 asthmatics and 34 suspected of asthma, age mean = 10.8 years, range = 6.0–16.0) underwent an incremental treadmill exercise test. Spirometry was performed at baseline and 1, 5-, 10-, 15-, and 20-min post exercise. Oscillometry was performed at baseline and at 3- and 18-min post exercise. Bronchodilator response to 200 µg albuterol was then assessed. EIB was defined as a forced expiratory volume in 1 s (FEV_1_) fall ≥10% from baseline. Expiratory and inspiratory resistance (Rrs) and reactance (Xrs), their *z*-score (Ducharme et al. 2022), and their mean within-breath differences (ΔRrs = Rrs_exp_-Rrs_insp_, ΔXrs =_ _Xrs_exp_-Xrs_insp_) were calculated. Receiver operating characteristic (ROC) curves and their areas (AUCs) were used to evaluate impedance parameters’ performances in classifying EIB.

**Results:**

Asthmatic patients developed EIB more frequently than those suspected of asthma [18/34 (52.9%) vs. 2/34 (5.9%), *p* < 0.001]. In the 20 subjects with EIB, Rrs_insp_, Rrs_exp_, Xrs_insp_, and Xrs_exp_ peaked early (3’), and remained steady except for Xrs_insp,_ which recovered faster afterward. ΔXrs widened 18 min following the exercise and reversed sharply after bronchodilation (BD) (−1.81 ± 1.60 vs. −0.52 ± 0.80 cmH_2_O × s/L, *p* < 0.001). Cutoffs for EIB leading to the highest AUCs were a rise of 0.41 in *z*-score Rrs_insp_ (Se: 90.0%, Sp: 66.7%), and a fall of −0.64 in *z*-score Xrs_insp_ (Se: 90.0%, Sp: 75.0%). Accepting as having “positive” postexercise oscillometry changes those subjects who had both *z*-scores beyond respective cutoffs, sensitivity for EIB was 90.0% (18/20) and specificity, 83.3% (40/48).

**Conclusion:**

Oscillometry parameters and their within-breath differences changed markedly in pediatric patients presenting EIB and were restored after the bronchodilator. Strong agreement between *z*-scores of inspiratory oscillometry parameters and spirometry supports their clinical utility, though larger studies are required to validate these findings in a broader population.

## Introduction

A common complaint in pediatric outpatients visiting the pulmonologist is the limitation of physical activity, which they often refer to as dyspnea, cough, chest pain, or other less-well-defined symptoms elicited by exercise. These patients embrace a heterogeneous group with some having a history of recurrent respiratory symptoms, while others refer to exercise limitation as their only complaint (1, 2). Consequently, children are less prone to get involved in sports with further physical and psychosocial drawbacks (1). Testing for exercise-induced bronchoconstriction (EIB) is suitable, even when an asthma diagnosis seems inconsistent with the clinical history (3–5). Conversely, the presence of EIB in yet-diagnosed asthmatic children suggests poor disease control (2, 3).

Classical exercise airway challenges entail repeated spirometry at baseline and in a timely manner after exercise, with skilled patients being able to perform these maneuvers. In children, oscillometry, which is also called the forced oscillation technique (FOT) needing quiet breaths, is feasible and reproducible (6, 7). Throughout input wave or pressure pulses at the mouth opening, oscillometry measures of respiratory resistance (Rrs) and reactance (Xrs) disclose contributions to the total respiratory impedance (Zrs) from proximal and peripheral airways and tissues, and their response to several bronchial stimuli (6, 8). Children tolerate well the midwave 8 Hz input frequency and several reference values for Rrs and Xrs at this frequency are now available (9–11).

A practical use of the oscillometry parameters is to compute them separately for the inspiratory and expiratory phases and assess their changes during the breath cycle. This approach is spreading as it may increase sensitivity and specificity to specific pathologies and conditions (6, 12). In particular, the within-breath difference between the mean inspiratory Xrs (Xrs_insp_) and the mean expiratory Xrs (Xrs_exp_) increases with the occurrence of tidal expiratory airflow limitation (13). This approach has been useful to assess adult patients with chronic obstructed pulmonary disease (COPD) and children with cystic fibrosis (13–15). In this regard, no studies in pediatric patients have sought to evaluate the within-breath changes in reactance during EIB. Also, previous studies did not consider changes in *z*-scores of impedance parameters, but absolute or relative changes were reported. The use of *z*-scores may better evaluate the effect of exercise.

We hypothesized that EIB [as assessed by the maximal forced expiratory volume in 1 s (FEV_1_) fall], other than inducing changes in oscillometry parameters, also induces wide within-breath differences in Xrs that lessen as the bronchial patency restores. In keeping, we aimed to assess the response in impedance parameters following an exercise challenge in patients reporting exercise-induced symptoms (EIS), either without or with a previous diagnosis of asthma. For this purpose, we assessed Rrs, Xrs, at 8 Hz, and their within-breath differences at baseline and after the exercise challenge.

## Materials and methods

### Subjects

We enrolled 73 consecutive outpatients attending the pulmonary section of our Pediatric Unit (Sant’Andrea Hospital, Rome, Italy) for EIS, to perform a bronchial challenge with exercise. After excluding five subjects not fulfilling the inclusion criteria (below), the remaining 68 patients yielded two groups of 34 subjects each as follows: (1) EIS without a previous diagnosis of asthma (“suspected asthma”), and (2) EIS and asthma (“asthma”). The suspected asthma group comprised new-attendant children reporting exercise limitation as their main complaint, often associated with rhinitis, without a clinical history of asthma. None of these patients had hoarseness, stridor, chocking, or abnormal spirometry (flattening of the inspiratory portion of the flow volume) suspected of exercise-induced laryngeal obstruction (EILO). The asthma group included patients whose diagnosis was based on both clinical history and pulmonary function (responsiveness to bronchodilators or to bronchial provocation tests) and had at least a partly controlled level of symptom control (16). For all subjects, inclusion criteria were a baseline FEV1 ≥80% of the predicted value, absence of respiratory infections in the previous 4 weeks, avoidance of inhaled corticosteroids, leukotriene antagonists, and antihistamines in the last 10 days, long-acting beta-agonists in the last 24 h, short-acting in the last 12 h. Parents signed a written informed consent; the Ethical Review Board of our Institution approved the study.

### Study design

During a single session, patients underwent a medical visit, and parents completed a questionnaire on respiratory health including current respiratory symptoms and asthma medications. The child yielded information on EIS such as cough, dyspnea, and chest pain/tightness. Patients followed skin prick testing, oscillometry, and the fraction of exhaled nitric oxide (FE_NO_) measurements in this order before baseline spirometry and exercise testing.

### Skin prick tests

Sensitization to common inhaled and food allergens was tested by skin prick tests (SPTs) (ALK-Abellò, Milan, Italy) including house dust mites (Dpt and Dpf), molds (Alternaria alternata), dog and cat fur, Parietaria judaica, mixed grass pollens, mixed tree pollens, egg, and milk. Positive and negative controls were Histamine 0.1 mg/mL and glycerol solution, respectively. Positive SPTs were defined by a wheal size of at least 3 mm after subtraction of the negative control.

### Oscillometry

Impedance measurements were obtained by oscillometry at a mono frequency of 8 Hz using the Resmon Pro Full device (ResTech, Milano, Italy). Children were seated, connected to the bacterial filter mouthpiece (DAR^™^, Covidien IIc, Mansfield, USA), and breathed normally wearing a nose clip whereas the technician supported their cheeks. Three baseline replicates from at least 10 breaths each, free of artifacts, with a coefficient of variance (CV) ≤15% for Rrs were accepted (6). Tidal volume (Vt), respiratory rate (RR), and minute ventilation (V’E) for each set of measurements were also recorded. Expiratory and inspiratory Rrs and Xrs were expressed both as absolute means and as *z*-scores using reference values derived from the same model of device that we used (11) according to recent recommendations for technical standards in oscillometry measurements (6). Impedance modulus (Zrs) was also computed for both inspiratory and expiratory phases. Mean within-breath differences (Rrs_exp_-Rrs_insp_, Xrs_exp_-Xrs_insp_) were calculated and reported as ΔRrs and ΔXrs, respectively.

### FE_NO_

FE_NO_ was measured with an electrochemical device (HyPair FENO, Medisoft Group, Sorinnes, Belgium). Children did single-breath maneuvers at constant expiratory pressure and flow of 50 mL/s as recommended. Mean values from two measurements agreeing within 10% from at least three expiratory maneuvers were calculated (17).

### Spirometry

Spirometry was performed with a Quark PFT (Cosmed Srl, Rome, Italy) according to the ATS/ERS guidelines (18). The best FEV_1_ from three measurements was recorded. Dynamic volumes and flows were both percentages and *z*-scores of the predicted values (19).

### Exercise testing

After baseline oscillometry and spirometry, patients underwent an incremental treadmill exercise test (20). Running was set at 6 km/h with a pendant of 10% until children reached the target heart rate of over 85% of the predicted maximum, calculated as 220-age years (21). Room temperature (20–24°C) and relative humidity (50%–60%) were kept stable during the procedure. After the exercise, subjects repeated spirometry at 1, 5, 10, 15, and 20 min, and oscillometry at 3 and 18 min. The lowest FEV_1_ was taken to calculate its decline from the baseline; EIB was defined as the fall in FEV_1_ ≥10% from baseline after exercise (4).

Following the 20-min spirometry, subjects received albuterol 200 µg (MDI with spacer); after a 15 min interval, they repeated oscillometry and spirometry, in this order.

Postexercise changes in impedance from baseline were calculated and reported depending on the parameter: the maximal rise in Rrs or the maximal decrease in Xrs from their respective baseline values. The changes were both percentages from baseline and differences from baseline *z*-scores. To calculate differences, *z*-scores for impedance parameters at every postexercise step were computed; then the *z*-scores for postexercise values were subtracted from their respective baseline values.

### Statistical analysis

Continuous variables were assessed for normal distribution (K-S test); pulmonary function variables were expressed as both means ± SD and *z*-scores using reference values for spirometry (19) and impedance (11); categorical variables were given as numbers and percentages. Postexercise changes and bronchodilator responses were expressed as medians and interquartile ranges (IQRs). The Mann–Whitney *U* test was used for unpaired comparisons and the non-parametric ANOVA Friedman with *post hoc* Dunnett's test to assess differences between postexercise and baseline measurements. The Wilcoxon test was used for paired comparisons between postbronchodilator and last postexercise measurements.

Contingency tables (*χ*^2^ with Fisher's correction) were applied to compare categorical variables. Receiver operating characteristic (ROC) curves were constructed to analyze the relationship between sensitivity (Se) and 1-specificity (Sp) for impedance measurements in classifying EIB. The areas (AUC) under ROC curves were computed and used to identify the oscillometry parameters that better classified EIB. The cutoff values were determined by maximizing the Youden index. We also computed the cutoff values that maximize the Youden index among the ones providing Sp >Se. Spearman's Rho tests were used to assess correlations between continuous variables. The SPSS software (Version 27; SPSS Inc., Chicago, IL, USA) was used for statistical analyses. Two-tailed *p* values of <0.05 were regarded as statistically significant.

## Results

Sixty-eight patients reporting EIS (age 10.8 ± 2.6 years, range 6.0–16.0, 41 males) completed all measurements and were allocated in two subsets of 34 subjects each (suspected asthma, and asthma). The asthmatic patients had more frequent cigarette smoke exposure at home, atopy, use of inhaled corticosteroids, and slightly lower FEV_1_/forced vital capacity (FVC) and FEF_25__–75_ but no different FEV_1_, FeNO, or impedance measurements than patients suspected of asthma (Table 1). Following the exercise, the asthmatic patients presented EIB more frequently than the suspected asthmatics [18/34 (52.9%) vs. 2/34 (5.9%), *p* < 0.001]. The pooled group of patients reporting EIS consisted of 20/68 patients with EIB (29.4%) and 48/68 (70.6%) without EIB.

**Table 1:** Characteristics of the patients reporting exercise-induced symptoms (EIS).

Variables	Suspected asthma (*n* = 34)	Asthma (*n* = 34)
Gender, M/F	17/17	24/10
Age, years	10.8 ± 2.5	10.8 ± 2.7
Weight, kg	43.8 ± 12.5	44.0 ± 12.6
Height, cm	145.8 ± 15.1	144.9 ± 13.7
BMI percentile	74.0 ± 25.0	72.8 ± 26.0
Reported EIS		
Cough	21 (61.8)	14 (41.2)
Dyspnea	24 (70.6)	24 (70.6)
Chest pain	1 (2.9)	7 (20.6)
Other EI-symptoms	7 (20.6)	1 (2.9)
Clinical data		
Rhinitis	22 (64.7)	24 (70.6)
Parents smoke at home	6 (17.6)	17 (50.0)^§^
Atopy	24 (70.6)	32 (94.1)^§^
FeNO, ppb	31.6 ± 24.2	31.3 ± 23.9
Therapy last 12 months		
Inhaled corticosteroids	19 (55.9)	30 (88.2)*
Antileukotrienes	8 (23.5)	9 (26.5)
Antihistamines	16 (47.1)	15 (44.1)
Pulmonary function		
FEV_1_%	100.7 ± 9.6	97.0 ± 10.4
FEV1 *z*-score	0.06 ± 0.82	−0.26 ± 0.89
FVC%	95.0 ± 11.6	95.9 ± 9.0
FVC *z*-score	−0.43 ± 0.98	−0.35 ± 0.77
FEV_1_/FVC%	105.8 ± 7.6	100.7 ± 8.3^§^
FEV1/FVC *z*-score	1.05 ± 1.26	0.22 ± 1.24*
FEF_25−75_%	106.9 ± 20.2	92.9 ± 22.1^§^
FEF_25−75_ *z*-score	0.26 ± 0.87	−0.32 ± 0.91*
Rrs_insp_,cmH_2_O/L/s	4.79 ± 1.66	5.26 ± 1.44
Rrs_insp_ *z*-score	0.26 ± 1.08	0.75 ± 1.18
Rrs_exp_,cmH_2_O/L/s	5.57 ± 2.08	6.00 ± 1.93
Rrs_exp_ *z*-score	0.36 ± 1.15	0.72 ± 1.30
ΔRrs, cmH_2_O/L/s	0.78 ± 0.58	0.74 ± 0.86
Xrs_insp_, cmH_2_O/L/s	−0.72 ± 0.44	−0.93 ± 0.85
Xrs_insp_ *z*-score	0.41 ± 0.76	0.09 ± 1.23
Xrs_exp_, cmH_2_O/L/s	−1.12 ± 0.76	−1.54 ± 1.17
Xrs_exp_ *z*-score	0.34 ± 0.68	−0.17 ± 0.20
ΔXrs, cmH_2_O/L/s	−0.40 ± 0.63	−0.61 ± 0.83
Zrs_insp_, cmH_2_O/L/s	4.86 ± 1.69	5.38 ± 1.54
Zrs_exp_, cmH_2_O/L/s	5.70 ± 2.17	6.24 ± 2.13

Variables are expressed as numbers (percentages) or means (SD). Prick index: sum of positive allergen skin-wheal reactions. Rrs, Xrs, and Zrs: respiratory resistance, reactance, and impedance modulus, respectively, measured during inspiratory (insp) and expiratory (exp) phases. Δ: the within-breath difference between expiratory and inspiratory resistance (ΔRrs), and reactance (ΔXrs).

* *p* < 0.01 vs. subjects suspected of asthma.

^§^ *p* < 0.05.

### Postexercise ventilatory changes and oscillometry

Vt (L) increased after exercise among patients with EIB (baseline: 0.67 ± 0.26, 3’: 0.80 ± 0.36, 18’: 0.77 ± 0.33, *p* = 0.029) but not in patients without EIB (0.64 ± 0.26, 0.71 ± 0.29, and 0.66 ± 0.27, respectively, *p* = 0.226). By contrast, RR (beats/min) remained stable in both groups, resulting in a non-significative increase in V’E (L/min) from baseline in both patients with EIB (baseline: 15.7 ± 5.0, 3’: 18.0 ± 7.0, 18’: 16.8 ± 4.5, *p* = 0.250) and without EIB (16.2 ± 5.2, 17.5 ± 6.0, and 16.2 ± 5.3, respectively, *p* = 0.610). In the pooled population, ventilation changes from baseline did not correlate with those in oscillometry at 3’post exercise, though, at 18’ post exercise, the increases in Vt and V’E slightly correlated with the fall in the Xrs_exp_ *z*-score (*ρ*=−0.31 and *ρ*=−0.30, respectively, *p* < 0.05).

### Lung function by EIB outcomes

Subjects with EIB had lower baseline FEV_1_ and higher Rrs_insp_ than subjects without EIB (FEV_1_%: 94.9 ± 9.8 vs. 100.4 ± 9.9; Rrs_insp_ cmH_2_O/L/s: 5.71 ± 1.36 vs. 4.74 ± 1.56, *p* < 0.05 for both comparisons). The differences among groups were more evident considering *z*-scores of baseline oscillometry parameters (EIB vs. without EIB: *z*-score Rrs_insp_, 1.18 ± 1.02 vs. 0.22 ± 1.08, *p* < 0.01; *z*-score Xrs_exp_, −0.43 ± 1.26 vs. 0.30 ± 0.79, *p* < 0.05).

**Figure 1:**
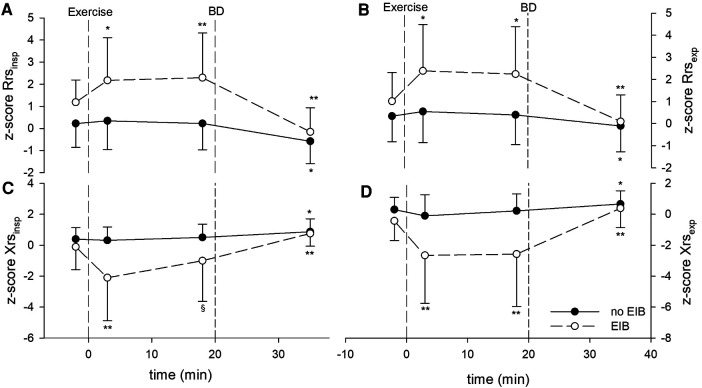
*Z*-scores of impedance parameters measured at baseline, after the exercise challenge, and after bronchodilation (BD) in patients with exercise-induced bronchoconstriction (EIB) and those without. (**A**) *z*-score Rrs_insp_, (**B**) *z*-score Rrs_exp_, (**C**) *z*-score Xrs_insp_, and (**D**) *z*-score Xrs_exp_. *p < 0.01 and **p < 0.001 for postexercise measurements (3 and 18 min) vs. baseline, or BD vs. 18 min after exercise.

**Figure 2:**
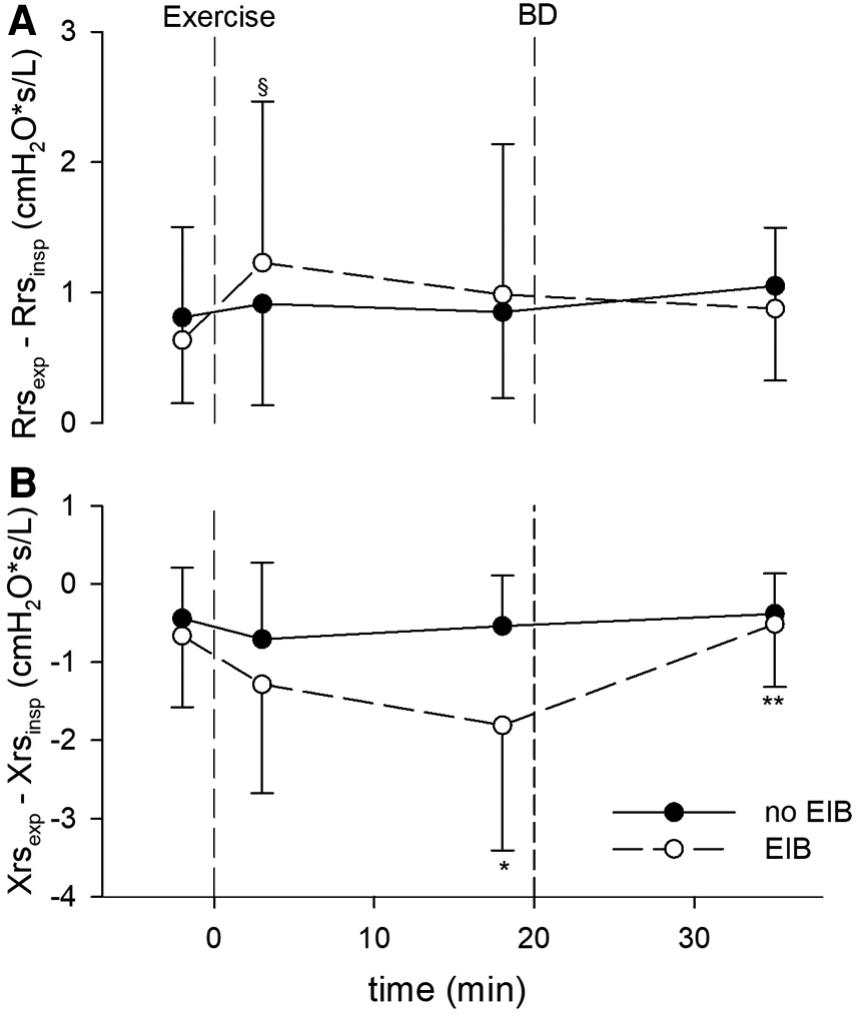
(**A**) Within-breath difference between expiratory and inspiratory resistance (ΔRrs), cmH_2_O/L/s, measured at baseline, after the exercise challenge, and after bronchodilation (BD) in patients with exercise-induced bronchoconstriction (EIB) and those without. ^§^*p* < 0.05 vs. baseline. (**B**) Within-breath difference between expiratory and inspiratory reactance (ΔXrs), cmH_2_O/L/s, at the same intervals. *p < 0.01 vs. baseline; **p < 0.001 vs. 18 min after exercise.

Postexercise Rrs, Xrs, ΔRrs, and ΔXrs remained almost unchanged from baseline in patients without EIB but changed markedly in those with EIB shortly after exercise. In subjects with EIB, Rrs_insp_, Rrs_exp_, Xrs_insp_, and Xrs_exp_ peaked early (3’), and remained quite steady except for Xrs_insp_, which tended to recover afterward (Figure 1). Consequently, ΔXrs fell markedly 18 min following the exercise (Figure 2). Also, in EIB patients, ΔXrs recovered sharply after bronchodilation (BD) (−1.81 ± 1.60 vs. −0.52 ± 0.80 cmH_2_O × s/L, *p* < 0.001). Maximal changes in both spirometry and oscillometry measurements after exercise and after bronchodilation clearly divided subjects with vs. without EIB (Table 2).

**Table 2:** Maximal postexercise changes and bronchodilator responses (BDR) by EIB outcomes.

Variables	Without EIB (*n* = 48)		EIB (*n* = 20)	
Postexercise	% change	*z*-score change	% change	*z*-score change
Fall in FEV_1_	−2.5 (−5.3, 0.0)	−0.21 (−0.45, 0.0)	−14.9 (−21.4, −10.5)**	−1.21 (−1.69, −0.89)**
Fall in FEF_25−75_	−8.4 (−13.5, −1.27)	−0.33 (−0.53, −0.06)	−26.9 (−37.1, −17.5)**	−0.85 (−1.28, −0.53)**
Rise in Rrs_insp_	3.7 (−2.9, 10.2)	0.18 (−0.15, 0.58)	15.3 (9.5, 24.8)**	0.87 (0.51, 1.44)**
Rise in Rrs_exp_	5.3 (−5.4, 13.3)	0.28 (−0.23, 0.75)	25.9 (3.4, 50.4)*	1.36 (0.22, 2.68)*
Fall in Xrs_insp_	−15.1 (−63.3, 19.4)	−0.26 (−0.74, 0.38)	−96.7 (−221.0, −46.0)**	−1.66 (−2.89, −1.05)**
Fall in Xrs_exp_	−36.2 (−85.9, 14.0)	−0.38 (−0.84, 0.23)	−74.0 (−230.2, −35.7)*	−1.14 (−5.23, −0.57)**
Rise in Zrs_insp_	3.7 (−2.6, 11.5)	–	19.3 (11.2, 28.1)**	–
Rise in Zrs_exp_	6.6 (−4.5, 20.3)	–	27.9 (6.8, 72.0)*	–
BDR				
Rise in FEV_1_	3.43 (0.10, 6.2)	0.33 (0.01, 0.54)	14.3 (7.2, 27.6)**	1.13 (0.56, 1.48)**
Rise in FEF_25−75_	12.4 (5.1, 20.6)	0.53 (0.18, 0.80)	57.6 (33.3, 73.5)**	1.37 (0.97, 1.86)**
Fall in Rrs_insp_	−15.5 (−24.7, −8.0)	−0.77 (−1.22, −0.32)	−33.9 (−45.0, −22.1)**	−2.25 (−2.84, −1.45)**
Fall in Rrs_exp_	−9.9 (−22.1, 3.3)	−0.48 (−1.00, 0.14)	−30.9 (−39.3, −18.8)**	−1.89 (−2.80, −1.21)**
Rise in Xrs_insp_	33.3 (−3.0, 56.0)	0.32 (−0.03, 0.73)	55.9 (35.1, 65.8)^§^	1.07 (0.65, 2.60)**
Rise in Xrs_exp_	26.2 (−3.3, 55.5)	0.36 (−0.05, 0.71)	62.6 (41.0, 69.7)*	1.81 (0.76, 4.59)**
Fall in Zrs_insp_	−15.3 (−23.3, −7.8)	–	−34.1 (−45.5, −22.6)**	–
Fall in Zrs_exp_	−10.4 (−21.7, 3.1)	–	−33.0 (−42.8, −22.6)**	–

EIB: exercise-induced bronchoconstriction. Rrs, Xrs, and Zrs: respiratory resistance, reactance, and impedance modulus, respectively, measured during inspiration (insp) and expiration (exp). Maximal postexercise fall (or rise) changes from baseline are shown as both percentages and differences between *z*-scores at the respective steps. Bronchodilator responses were calculated from the last postexercise measurement. Variables are expressed as medians and interquartile ranges (IQRs).

* *p* < 0.01.

** *p* < 0.001 vs. subjects without EIB.

^§^ *p* < 0.05.

### Identifying EIB by oscillometry

In the pooled population, maximal percentage impedance changes correlated slightly better with maximal percentage changes in FEV_1_ than those in FEF_25–75_ after exercise, while the opposite was true after bronchodilation. All correlations improved by using maximal changes in Rrs and Xrs *z*-scores (Table 3).

**Table 3:** Spearman's correlations between maximal changes in impedance and spirometry parameters after exercise testing and following bronchodilator administration in the pooled population (*n* = 68).

Exercise	% fall in FEV_1_		% fall in FEF_25−75_	
	r	p	r	p
% rise in Rrs_insp_	−0.52	<0.001	−0.48	<0.001
% rise in Rrs_exp_	−0.35	0.003	−0.26	0.030
% fall in Xrs_insp_	0.38	0.001	0.36	0.002
% fall in Xrs_exp_	0.30	0.014	0.30	0.013
% rise in Zrs_insp_	−0.57	<0.001	−0.51	<0.001
% rise in Zrs_exp_	−0.37	0.002	−0.31	0.010
Rise in *z*-score Rrs_insp_	−0.57	<0.001	−0.53	<0.001
Rise in *z*-score Rrs_exp_	−0.36	0.003	−0.32	0.009
Fall in *z*-score Xrs_insp_	0.54	<0.001	−0.49	<0.001
Fall in *z*-score Xrs_exp_	0.41	<0.001	0.43	<0.001
Bronchodilator	% rise in FEV_1_		% rise in FEF_25−75_	
	r	p	r	p
% fall in Rrs_insp_	−0.51	<0.001	−0.62	<0.001
% fall in Rrs_exp_	−0.50	<0.001	−0.60	<0.001
% rise in Xrs_insp_	0.20	0.103	0.23	0.067
% rise in Xrs_exp_	0.34	0.005	0.36	0.003
% fall in Zrs_insp_	−0.52	<0.001	−0.63	<0.001
% fall in Zrs_exp_	−0.51	<0.001	−0.60	<0.001
Fall in *z*-score Rrs_insp_	−0.57	<0.001	−0.66	<0.001
Fall in *z*-score Rrs_exp_	−0.53	<0.001	−0.62	<0.001
Rise in *z*-score Xrs_insp_	0.41	<0.001	0.44	<0.001
Rise in *z*-score Xrs_exp_	0.51	<0.001	0.54	<0.001

Rise (or fall) in *z*-scores was calculated as the difference between the maximal postexercise Rrs *z*-score (or the minimal postexercise Xrs *z*-score) and the respective baseline *z*-score. Bronchodilation was calculated as the difference between the postbronchodilator *z*-score and the postexercise (18 min) *z*-score for each oscillometry parameter. Postexercise and bronchodilator responses are also shown as percentage changes in respiratory resistance (Rrs), reactance (Xrs), and impedance modulus (Zrs), both during inspiration (insp) and expiration (exp).

**Figure 3:**
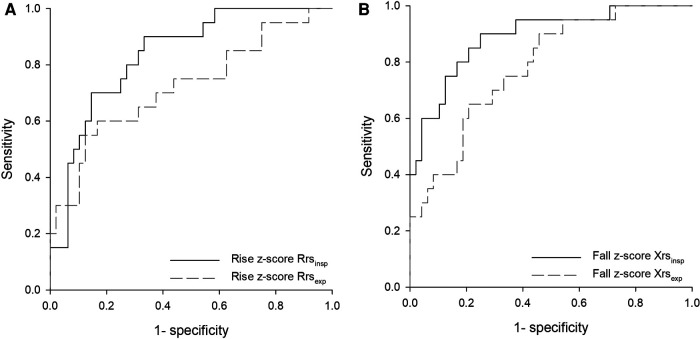
Receiver operating characteristic (ROC) curves for the postexercise change of respiratory impedance parameters measured at 8 Hz as predictors of exercise-induced bronchoconstriction (EIB). (**A**) Rise in *z*-scores of inspiratory and expiratory resistance (Rrs_insp_, Rrs_exp)_, areas under curves (AUCs): 0.84, *p* < 0.001; 0.72, *p* < 0.005. (**B**) Fall in *z*-scores of inspiratory and expiratory reactance (Xrs_insp_, Xrs_exp)_, AUCs 0.89 and 0.78, respectively; *p* < 0.001 for both.

AUCs-ROC were higher when oscillometry parameters were expressed as *z*-scores. Inspiratory parameters resulted in better classification performances (Figure 3). Maximized Youden index cutoffs for EIB of oscillometry parameters leading to the highest AUC are reported in Table 4. More stringent cutoffs for impedance changes, i.e., providing Sp>Se, were an increase of 0.67 in *z*-score Rrs_insp_ (Se: 70.0%, Sp: 85.4%), a fall of −1.14 in *z*-score Xrsinsp (Se: 80.0%, Sp: 83.3%), and a 15.3% increase in Zrs_insp_ (Se: 75%, Sp: 85.4%).

**Table 4:** EIB (fall in FEV_1_ ≥10%) by cutoff values of impedance for all the patients in the group (*n* = 68).

Parameter		AUC (CI)	Cutoff	Sensitivity (%)	Specificity (%)	PPV* (%)	NPV* (%)
Rise in Rrs_insp_	%	0.80 (0.70–0.91)	8.88	80.0	72.9	55.2	89.7
	*z*-score	0.84 (0.74–0.94)	0.41	90.0	66.7	52.9	94.1
Fall in Xrs_insp_	%	0.78 (0.65–0.90)	−29.9	95.0	58.3	48.7	96.6
	*z*-score	0.89 (0.80–0.98)	−0.64	90.0	75.0	60.0	94.7
Rise in Zrs_insp_	%	0.85 (0.75–0.94)	15.29	75.0	85.4	68.2	89.1
	cmH_2_O × s/L	0.86 (0.77–0.95)	0.46	90.0	72.9	58.1	94.6
Widest ΔXrs postexercise	cmH_2_O × s/L	0.76 (0.63–0.89)	−1.16	65.0	85.4	65.0	85.4

Rise (or fall) in *z*-scores: Difference between the maximal postexercise Rrs *z*-score (or the minimal postexercise Xrs *z*-score) and their respective baseline *z*-score. ΔXrs: Difference between mean expiratory and inspiratory reactance (cmH_2_O × s/L) measured 18 min after the exercise challenge. PPV, positive predictive values; NPV, negative predictive values.

* PPV and NPV are computed considering the EIB incidence of our study (29%).

### Postexercise outcome differences according to oscillometry and spirometry

**Figure 4:**
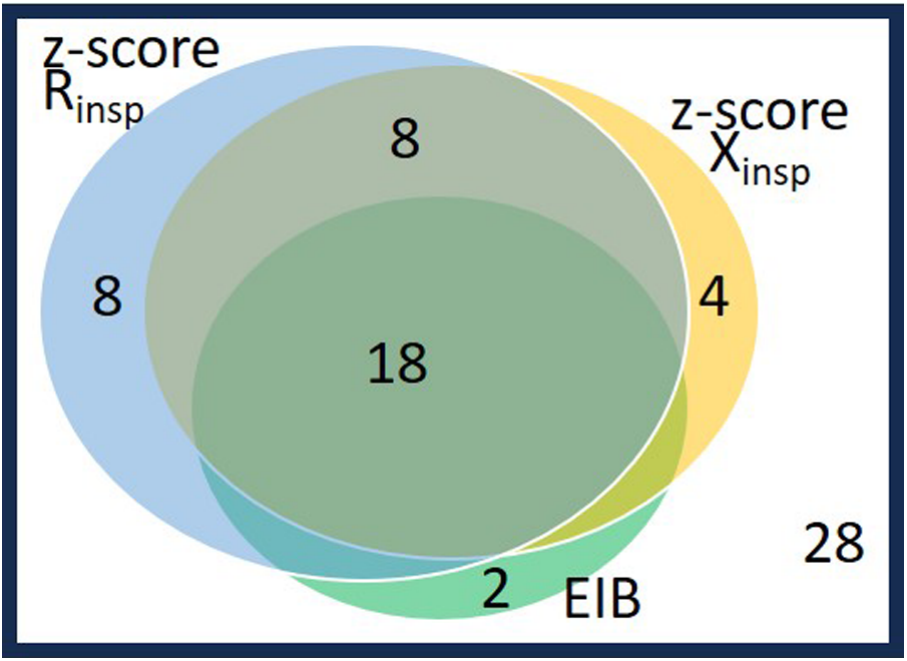
Venn diagram on the 40 patients with abnormal postexercise changes as defined either by spirometry or by oscillometry: 20 patients with EIB (fall in FEV_1_ ≥10%) and 20 patients without EIB and impedance changes beyond their cutoffs (*n* = 8: rise in Rrs_insp_ *z*-score>0.41; *n* = 4: fall in *z*-score Xrs_insp_>−0.64; *n* = 8: both changes in Rrs_insp_ and Xrs_insp_ *z*-scores are abnormal). The other 28 patients (lower right corner), did not change by spirometry nor by oscillometry. See also maximized Youden index cutoffs for EIB of oscillometry parameters in Table 4.

The relationship between EIB defined by spirometry and changes in *z*-scores of inspiratory oscillatory parameters above the cutoffs maximizing the Youden index is described in Figure 4.

Approximately 90% (18 subjects including 12 males) of the 20 spirometry-defined EIB subjects responded to exercise according to both *z*-scored Rrs_insp_ and Xrs_insp_, whereas 10% (two boys) did not respond above these *z*-score cutoffs. Conversely, among the 48 subjects without EIB, eight (16.7%, all males) subjects responded to exercise according to both *z*-score Rrs_insp_ and *z*-score Xrs_insp_, and 12 subjects (25.0%, nine males) responded in accordance to either *z*-score Rrs_insp_ or *z*-score Xrs_insp_. These two subgroups had similar baseline lung function and body mass index (BMI) percentile to the remaining 28 (58.3%) non-responder subjects according to oscillometry and without EIB.

The eight subjects without EIB responding to exercise according to both oscillatory *z*-scores had larger bronchodilator responses (DFEV_1_: 7.0 ± 5.4 vs. 2.6 ± 4.0; DFEF_25–75_: 28.0 ± 19.7 vs. 10.5 ± 12.0, *p* < 0.05), male incidence (8/8 vs. 10/28, *p* < 0.001), but no different asthma incidence (4/8 vs. 9/28, *p* = 0. 422) than the 28 subjects not responding for both oscillometry and spirometry. The 12 subjects responding only for *z*-score Rrs_insp_ or *z*-score Xrs_insp_ did not differ from the non-responding group, except for a higher male incidence (9/12 vs. 10/28, *p* = 0.038).

Accepting as having “positive” postexercise oscillometry changes those subjects who had both *z*-scores beyond respective cutoffs (0.41 increase in *z*-score Rrs_insp_ and −0.64 fall in *z*-score Xrs_insp_), sensitivity for EIB was 90.0% (18/20) and specificity, 83.3% (40/48).

## Discussion

Our findings support the utility of impedance measurements following an exercise challenge in outpatients reporting EIS. The occurrence of EIB raised Rrs, decreased Xrs, and widened the within-breath difference in Xrs; such a difference reversed close to that baseline after bronchodilation with albuterol. To our knowledge, evaluating oscillometry parameters in terms of *z*-scores computed separately for inspiration and expiration and the within-breath analysis of reactance has not previously been described to assess the exercise challenge. Also, we added to the scarce data on exercise testing at 8 Hz (22), a suitable forcing frequency for the pediatric population.

Notwithstanding that exercise increases minute ventilation, preceding studies assessing breathing parameters in adults (23) and adolescents (24) have found uninfluential V’E (23) and changes in Vt, RR, and V’E on oscillometry measurements for assessing EIB. Neither did we find a relationship between changes in ventilation and those in oscillometry early after exercise; still, the rise in Vt and V’E did correlate slightly with the fall from baseline in Xrs_exp_ (but not with Xrs_insp_) 18 min after the exercise ended.

### Oscillometry by EIB outcomes

Several studies have reported changes in respiratory impedance following exercise in subjects with asthma or asthma-like symptoms including young adults (23, 25, 26), children, adolescents (22, 24, 27–31), and preschool children (32, 33). These studies showed larger dynamic impedance changes from the few minutes following exercise in subsets of asthmatic patients or wheezy children than in control subjects, selected either as healthy (23, 33) or without ongoing airway hyperresponsiveness (26).

In our population of subjects reporting EIS, changes in oscillometry were not statistically significant in subjects without EIB. This is not unexpected, as there is a consistent percentage of subjects reporting EIS whose response to exercise cannot be objective by lung function tests (24, 34). BD improved oscillometry parameters in this group; however, improvements remained within the cutoff for BD response (relative percent change and *z*-score change) of healthy children aged 2–13 years (10).

Time changes in oscillometry parameters in EIB (spirometry assessed) subjects reported by previous studies (23, 24, 26, 27) showed an early worsening (at 5 min) of both Rrs and Xrs after exercise and a faster Xrs recovery than of Rrs. This suggests an earlier functional recovery of less proximal airways than of the relatively proximal airways. Our data hold up the early change in impedance parameters as shortly as 3 min after exercise and the slower Rrs recovery than Xrs in EIB subjects.

Both Rrs_insp_ and Rrs_exp_ remained high after 18 min post exercise. Xrs_insp_ gradually recovered while Xrs_exp_ remained persistently decreased, i.e., the Xrs recovery was supported from Xrs_insp_ only. This implies that the within-breath difference (ΔXrs) increased from baseline to the late postexercise step. Xrs reflects communicating lung volume and compliance (35) and is affected by ventilation heterogeneity. It changes minimally during tidal breathing in healthy subjects while it may drop sharply during expiration in subjects with tidal peripheral airway collapse (35, 36). Our data suggest that ΔXrs widens with acute airway obstruction, mostly peripheral, following exercise. These may be the results of different mechanisms. The development of flow limitation in some airways (13), the lower elastic recoil, and higher collapsibility of the small airways at lower volumes (37) leads to higher ΔXrs both directly and indirectly by increasing heterogeneity during expiration. Our data showed that increased ΔXrs was not the result of a further Xrs_exp_ decrease but of a faster Xrs_insp_ recovery from the early postchallenge step fall. Measuring within-breath Xrs components may provide additional information for the analysis of changes in lung mechanics after exercise.

### Identifying EIB by oscillometry

Changes in spirometry and oscillometry parameters were correlated but not totally overlapped.

Changes in *z*-scores rather than percentages from the baseline better assessed postexercise and postbronchodilator changes as detected by spirometry. This was especially verified for Xrs as drawbacks from using percentages of Xrs are especially evident when values close to zero are recorded (6).

Changes in *z*-scores of inspiratory rather than in expiratory oscillometry parameters best predicted EIB. Therefore, we agree with other studies reporting that inspiratory impedance parameters indicate EIB better than expiratory (24, 29). Conversely, in school-age children born extremely preterm, inspiratory impedance did not mirror EIB, suggesting a distinct prematurity-driven airway reactivity (38).

Percentage changes from baseline of Zrs_insp_ performed best against spirometry (Tables 3, 4), supporting the idea that this parameter, including contributions of both Rrs_insp_ and Xrs_insp_ changes, can better describe the changes in lung condition (24). However, reference equations are not available for Zrs_insp_ (and their intrabreath components) yet. Therefore, we cannot verify if using *z*-score Zrs_insp_ can further improve agreement with spirometry. The maximum postexercise widening of ΔXrs was also related to EIB even if it provided lower AUC than postexercise changes in *z*-scores of Rrs_insp_ or Xrs_insp_. Reference values of ΔXrs are not available. Reporting ΔXrs as a *z*-score may improve the correlation with spirometry parameters when assessing responses to the exercise challenge.

Previous cutoffs for oscillometry with spirometry-based bronchial challenges in children refer to the methacholine testing, which (using different forced frequencies and devices) varies from a 45%–70% increase in Rrs and a 50%–80% drop in Xrs (6). Instead, cutoffs for oscillometry with exercise testing are less well established. A recent study described a 27% increase in R5 and a 47% decrease in X5 as predictors of EIB, based on a fall in FEV_1_ ≥10% in asthmatic adults (23). Another study disclosed an increase of 0.035 kPa/L/s in R5 after 5 min of exercise (and a 0.055 kPa/L/s as maximum increase) to distinguish asthmatic patients with vs. those without airway hyperresponsiveness to methacholine (26). The most recent study, in asthmatic children, reported a 14.1% increase in R5 within 30 min post exercise as a diagnostic of EIB (31). Differently, among adolescents with or without reported exercise-induced dyspnea, cutoffs were drawn from 95% oscillometry changes in those regarded as healthy. Such an approach identified *z*-score increases in Rrs of 0.68 and Xrs of 1.76 as cutoffs for EIB (24). Our cutoff providing Sp>Se for *z*-score Rrs_insp_ is close to the one determined on healthy children (0.67 vs. 0.68), though our *z*-score Xrs_insp_ at 8 Hz is lower than their change in Xrs5 (1.14 vs. 1.76), as expected using higher stimulation frequencies and considering only the inspiratory phase.

### Postexercise outcome differences according to oscillometry and spirometry

There is increasing evidence that spirometry and oscillometry are not always in accordance as they describe lung mechanics under different conditions (6, 12, 37). Spirometry requires a high lung volume maneuver and FEV_1_ reflects mainly airflow resistance of proximal airways (39). Oscillometry requires tidal breaths at resting lung volume, is also sensitive to peripheral airways, and is deemed complemental to spirometry for assessing airway responsiveness (6, 12). Changes in inspiratory Rrs well document the attenuation effect of EIB in spirometry with deep inspirations in asthmatic children in contrast with healthy controls (29). Small degrees of airway narrowing in healthy children have been attributed to transient hyperemia of the airway wall leading to local edema (29), a mechanism that could explain why the impedance increased after exercise in some of our subjects without EIB.

The best agreement between postexercise changes in spirometry (EIB) and oscillometry was seen in subjects showing changes beyond their identified cutoffs in both *z*-scores of Rrs_insp_ and Xrs_insp_. This supports the idea of the importance of considering changes in both Rrs and Xrs and correcting for anthropometric factors. However, as expected the two techniques did not lead to identical results because of different physiological lung conditions during the measurements and different difficulties in performing the measurements (6–8, 12). Two subjects who developed EIB (both males with asthma) did not show large changes in either *z*-scores of Rrs_insp_ or Xrs_insp_. On the contrary, large changes in these two parameters occurred in eight male subjects without EIB who had evenly distributed asthma diagnoses (4:4).

Incongruent growth of the airway's caliber relative to the growth of lung parenchyma, known as airway dysanapsis (characterized by low FEV_1_/FVC despite normal FEV_1_ and FVC) is relevant in children (39, 40). Boys have smaller airways than girls proportional to their lung growth (40, 41); obesity is associated with airway dysanapsis in children with or without asthma, and this airway–lung incongruence is linked to worse asthma control (40). Our eight male subjects without EIB who had large changes in both *z*-scores of Rrs_insp_ and Xrs_insp_ yielded no different baseline lung function (including FEV_1_/FVC) nor BMI percentile than the remaining subjects without EIB. Because these eight subjects had more changes in inspiratory reactance than the other subjects without EIB, they could have peripheral airway obstruction unrevealed by spirometry. Gender differences in small airway physiology have been reported among asthmatic subjects, as larger methacholine-induced gas trapping was found in males than females (42). Our eight subjects also showed greater changes in spirometry than the non-responding subjects after BD. An alternative hypothesis is that these eight boys, having a high airway hyperresponsiveness, could be prone to smooth-muscle relaxation after deep inhalations from FVC maneuvers. If so, the effect of exercise is more evident during quiet breathing and easily identified by oscillometry as the maneuver required for spirometry partially reverses the changes in lung mechanics.

### Limitations

We acknowledge the following limitations. We involved outpatients attending the pulmonology service for EIS in a real-life approach, thus, we are cautious about comparing results with other settings that also involve healthy controls. Still, oscillometry parameters drive their use for screening patients with EIS in similar outpatient settings. The mono frequency of 8 Hz that we used allows the exploration of both proximal and less proximal airways; thus, the changes in Rrs_insp_ and Xrs_insp_ evinced the involvement of different airway segments during EIB in our study.

Physiological ventilatory responses to exercise could influence our oscillometry measurements. However, the expiratory reactance was the sole parameter slightly influenced by Vt whereas no inspiratory impedance components, i.e., those better identifying EIB, correlated with the ventilatory parameters.

The broncho-motor effect of deep inspiration with forced expiratory maneuvers could also modify our measures (29, 43); to minimize this effect we did oscillometry maneuvers before those of spirometry. However, repeated maximal inspiratory maneuvers after the exercise challenge might result in broncho-protection leading to underestimating EIB, especially in asthmatic subjects whose response to deep inspirations depends on the degree of airway obstruction (43).

Our subjects ran without a facemask, despite it being preferred to inhale medical dry air for the exercise testing protocol (3, 4); this might decrease the number of positive test outcomes. Because more than half of our asthmatic patients had EIB, the overall blunt effect of deep inhalations on the broncho-motor tone or the environmental conditions during the test was probably modest.

We calculated ΔRrs and ΔXrs as mean within-breath differences (13–15), rather than computing the difference between the end-expiratory and the end-inspiratory impedance (i.e., both at zero flow). Although this recently suggested approach (44) has been indicated as potentially more sensitive to changes due to its reduced susceptibility to changes in breathing flow, our data exhibited very limited dependence on breathing patterns, which further strengthens the credibility of our findings.

In conclusion, we found marked changes in oscillometry parameters and raised within-breath differences in reactance among pediatric outpatients with self-reported EIS presenting EIB. Baseline oscillometry values were restored after the bronchodilator. The high agreement of *z*-scores of inspiratory parameters with spirometry supports their use for clinical evaluation although larger studies are needed to confirm the validity of the identified cutoff in a more general population. More studies are needed to establish the clinical role of within-breath differences in impedance during EIB in these children.

## Data Availability

The original contributions presented in the study are included in the article/Supplementary Material, further inquiries can be directed to the corresponding authors.
